# P-1185. Targeting and Universal Congenital Cytomegalovirus Screening in a Neonatal Intensive Care Unit

**DOI:** 10.1093/ofid/ofae631.1369

**Published:** 2025-01-29

**Authors:** Adriana Gonzalez Torriente, Tara Randis, Claudia M Espinosa

**Affiliations:** University of Miami/Jackson Health Systems, Miami, Florida; University of South Florida, Tampa, Florida; MORSANI COLLEGE OF MEDICINE PEDIATRICS, Tampa, Florida

## Abstract

**Background:**

Congenital cytomegalovirus (cCMV) infection, confirmed by urine or saliva CMV PCR within 3 weeks of life, affects 0.5 % of live births in the United States. This number varies in different populations, and it is affected by socio-demographic characteristics. In Neonatal Intensive Care Units (NICUs), higher prevalence has been reported in preterm and low birth weight infants, but timing of screening remains a challenge without universal screening because only 10% of cCMV are symptomatic. Regardless of symptoms, infected infants are at increased risk of adverse outcomes such as hearing loss. Thus, we sought to determine the incidence and associated characteristics of cCMV infection using targeting and subsequently universal screening in our NICU population

**Figure:**
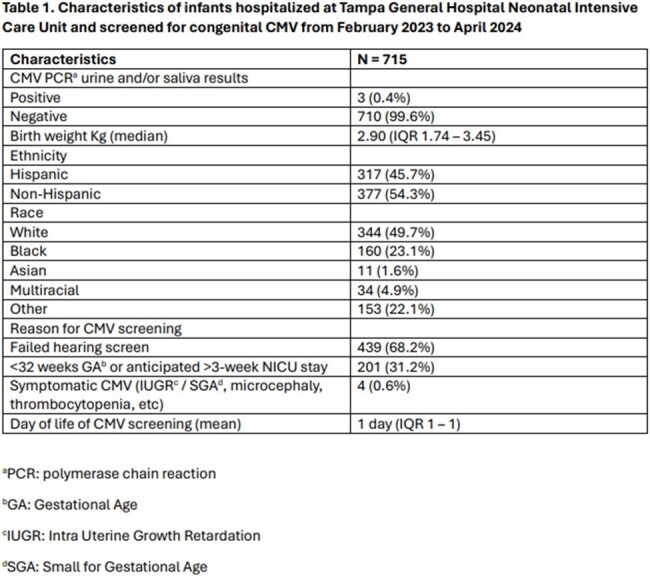

**Methods:**

From Feb 1, 2023 to Apr 30, 2024, infants who were admitted at Tampa General Hospital (TGH) NICU and had a CMV PCR testing performed during admission were followed prospectively. Before Feb 28, 2024, cCMV screening was performed only in infants who were symptomatic (IUGR, microcephaly, thrombocytopenia) or failed hearing screen. After that, all infants born preterm < 33 weeks gestational age (GA) and all infants with anticipated NICU stay > 3 weeks were screened for cCMV at admission. Characteristics such as GA, birth weight, and race and ethnicity are recorded in a RedCap database. Descriptive statistics were used

**Results:**

A total of 715 infants were screened for CMV while hospitalized in the TGH NICU during the study period. Most of the infants (68.2%) were screened after failing newborn hearing screen and only four (0.6%) were screened because of their symptoms. The characteristics of the infants with positive and negative saliva or urine CMV PCR are described in Table 1. Only three (0.4%) infants tested positive for CMV and were confirmed to have cCMV. Two infants with cCMV infection were screened because they were symptomatic

**Conclusion:**

At TGH NICU, the incidence of cCMV using targeting and universal screening was comparable with the U.S. general population and lower than reported in other NICUs. We plan to perform association when statistically feasible, and compare incidence of cCMV between the targeting and universal screening periods

**Disclosures:**

**Claudia M. Espinosa, MD, MSc**, AstraZeneca: Grant/Research Support|Clinetics: Grant/Research Support|Enanta: Grant/Research Support|Gilead: Advisor/Consultant|Jansen & Jansen: Advisor/Consultant|Kentucky Health Rural Association: Honoraria|Medimmune: Grant/Research Support|Melinta: Grant/Research Support|Merck: Grant/Research Support|Sanofi: Advisor/Consultant|Sanofi: Honoraria

